# Prevalence and correlates of diabetes and impaired fasting glucose among adults in Afghanistan: Insights from a national survey

**DOI:** 10.1177/20503121241238147

**Published:** 2024-03-20

**Authors:** Omid Dadras, Anita Nyaboke Ongosi, Chia-Wen Wang

**Affiliations:** 1 Department of Global Public Health and Primary Care, University of Bergen, Bergen, Norway; 2 International Institute of Socio-Epidemiology, Kyoto, Japan; 3 Saw Swee Hock School of Public Health, National University of Singapore and National University Health System, Singapore, Singapore; 4 Lloyd’s Register Foundation Institute for the Public Understanding of Risk, National University of Singapore, Singapore, Singapore

**Keywords:** Diabetes, impaired fasting glucose, risk factors, Afghanistan

## Abstract

**Objectives:**

Afghanistan is experiencing an escalating burden of noncommunicable diseases, with diabetes and impaired fasting glucose being of particular concern. To explore the prevalence of diabetes and impaired fasting glucose and associated factors among adult Afghans.

**Methods:**

This cross-sectional study used secondary data from a nationally representative survey, conducted in 2018 in Afghanistan. A blood sample was collected from the fingertip and tested through a strip to measure blood glucose. The complex sampling design and sampling weights were accounted for in all analyses to produce representative estimates of the target population in Afghanistan.

**Results:**

Of 3890 Afghan adults aged 18–69 years who participated in this survey, 11.07% and 10.32% had diabetes and impaired fasting glucose, respectively. For overweight individuals with abdominal obesity, the risk for diabetes became significantly elevated, with an adjusted relative risk ratio of 2.12 (95% CI: 1.10–4.09). However, the most pronounced effect was observed among individuals classified as having obesity with abdominal obesity with an adjusted relative risk ratio of 2.54 (95% CI: 1.37–4.70). Moreover, high cholesterol level was significantly associated with both impaired fasting glucose (ARRR: 2.52, 95% CI: 1.55–4.12) and diabetes (ARRR: 4.12, 95% CI: 2.59–6.56), whereas high blood pressure was significantly associated with only diabetes (ARRR: 1.82, 95% CI: 1.16–2.86).

**Conclusions:**

This investigation provides critical insight into the prevalence of diabetes and IFG among Afghan adults aged 18–69 years. Relative to the global average, the higher prevalence observed calls for specifically designed interventions targeting individuals with cardiometabolic risk factors, such as elevated body mass index, abdominal obesity, hypertension, and hypercholesterolemia.

## Introduction

Diabetes mellitus (DM) and impaired fasting glucose (IFG) are two major health concerns with significant global implications. According to the International Diabetes Federation, an estimated 463 million individuals worldwide had diabetes in 2019, and this number is projected to rise to 700 million by 2045.^1^ DM not only poses a substantial burden on healthcare systems but also contributes to increased morbidity and mortality rates owing to its association with various complications, such as cardiovascular diseases, kidney failure, and lower limb amputations.^2^

The prevalence of diabetes and IFG varies across different countries and regions, with higher rates reported in low- and middle-income countries (LMICs). Afghanistan, located in South Asia, is one such LMIC where the burden of diabetes is steadily increasing.^3^ Afghanistan faces numerous challenges in its healthcare system, including limited resources, political instability, and ongoing conflicts, further hindering the effective prevention and management of chronic diseases.^4^ Understanding the prevalence and identifying the correlates of diabetes and IFG in Afghanistan is crucial for developing targeted interventions and healthcare policies to address this growing public health issue.

Previous studies have reported varying prevalence rates of diabetes and IFG in Afghanistan. In 2012, a cross-sectional study conducted among adults aged ⩾40 years in Kabul, the capital city of Afghanistan, reported a prevalence rate of 13.2% for diabetes.^5^ Another study in 2015 reported a prevalence of 9.1% in Kabul.^6^ Similar studies conducted in Jalalabad^7^ and Herat,^8^ other major cities in Afghanistan, have reported a diabetes prevalence of 11.8% and 9.9% in 2013 and 2015, respectively. Although these studies provide important insights into the burden of diabetes in specific regions of Afghanistan, a comprehensive nationwide assessment is necessary to capture the full extent of the problem. The correlates of diabetes in Afghanistan are likely multifactorial, influenced by a complex interplay of genetic, environmental, and lifestyle factors. Limited physical activity, unhealthy dietary patterns, obesity, and low socioeconomic status have been identified as potential risk factors for diabetes in similar LMIC settings.^9–11^ However, the specific correlates in the Afghan population remain largely unexplored.

This study aims to provide a comprehensive overview of the prevalence and correlates of diabetes and IFG in Afghanistan. By synthesizing existing literature, we will identify the gaps in knowledge and highlight the need for further research to address this pressing public health issue. The findings from this study will serve as a foundation for policymakers, healthcare providers, and public health experts to develop evidence-based interventions and strategies for diabetes prevention and management in Afghanistan.

## Methods

### Study setting and participants

This is a cross-sectional study using secondary data from the World Health Organization (WHO) STEPwise approach to noncommunicable disease (NCD) risk factor surveillance (STEPS), a nationally representative survey, conducted in 2018 in Afghanistan.^12^ A nationally representative sample of adults aged 18–69 years was recruited through multistage cluster sampling. In the first stage, 55 of 417 districts were randomly selected as primary sampling units. In the second stage, villages or blocks were plotted as secondary sampling units, and the eligible households were listed and randomly selected. Finally, one person was randomly selected from each household. The total sample size was distributed proportionately to the size of the districts. The inclusion criteria in Afghanistan STEPS 2018 were: (1) Being household permanent residents aged 18–69; (2) Show willingness to participate in the study. The exclusion criteria were: (1) Temporary residents (are residing for less than 12 weeks) of households aged 18–69; (2) Household residents beyond the age limit; (3) Refuse to participate in the study.

### Sample size calculation

To ensure accurate estimates for each age and gender group using the survey data, the total numbers of three age-sex groups are considered when calculating the sample size. The STEPS proposed age groups follow the Global Burden of Disease (GBD) age categories, and we utilized the three age brackets per gender of 18–29, 30–44, and 45–69 years. The sample size for the six different population strata, consisting of three age groups for males and three age groups for females, was calculated using the below formula.

$$n=Z^{2}\;\frac{P(1-P)}{e^{2}}$$

With a 95% confidence level, 5% margin of error, 0.5 p, and 0.5 q, the resulting sample size was 384. To account for a 1.5 design effect and a 15% non-response rate, the sample size was adjusted to 662 for each age-sex group strata. The adjusted sample size of 662 was multiplied by the six age-sex groups (662*6) to arrive at a final sample size of 3972 household members. In total, 3890 eligible individuals aged 18–69 years completed the survey.

### Scales and measurement

The WHO STEPS wise approach was employed to collect data on sociodemographics, lifestyle, and behaviors by using the WHO STEPS instrument adapted for the Afghan adult population. The research team comprising trained healthcare professionals conducted anthropometric and blood test measurements in a controlled environment. In the first step, demographic (age, sex, education, number of households, etc.) and behavioral information (alcohol/tobacco consumption, daily fruit, and vegetable intake, etc.) were collected by a standard STEPs questionnaire translated into Pashto and Dari. In the second step, trained healthcare professionals, using a portable electronic weighing scale and inflexible bars, measured the participants’ weight, height, waist circumference, and blood pressure. In the final step, respondents’ blood samples were collected in a convenient setting from the fingertip and tested through a strip. The samples were used to measure blood glucose and total cholesterol levels using a dry chemistry (blood collection) method.^12^ To obtain accurate results, participants were informed and required to fast for at least 8 h before blood collection (12 h if cholesterol was measured). Since most of the blood samples were collected in the morning, participants were asked not to eat or drink (except plain water) from about 10 pm the night before. The blood collection usually took place in a convenient community setting, near participants’ homes. Portable android devices such as Cardiochek PA, Refloton Plus, Cholestech LDX are used in WHO’s STEPS to measure blood glucose and lipids by applying the blood collected from fingertip with a lancing device, and the results were reported in mg/dl.^13^ Diabetes was defined as a fasting plasma glucose level of ⩾126 mg/dl, insulin use, oral hypoglycaemic drug intake, or a prior diagnosis of diabetes.^14^ IFG was defined as a fasting plasma glucose level of 100–125 mg/dl^15^; a high cholesterol level was defined as a total cholesterol level of ⩾200 mg/dl.^16^ Body mass index (BMI) was calculated and categorized as underweight (<18.5 kg/m^2^), normal weight (18.5–24.9 kg/m^2^), overweight (25–29.9 kg/m^2^), and obese (⩾30 kg/m^2^) according to WHO recommendations.^17^ Abdominal obesity was defined as a waist circumference of >102 cm for men and >88 cm for women.^18^ Participants’ blood pressure was assessed using a calibrated sphygmomanometer, with hypertension defined as a systolic blood pressure (BP) of ⩾140 mmHg and/or diastolic BP of ⩾90 mmHg or in case the participant was on antihypertensive medication.^19^ Before measuring the BP, participants were asked to sit quietly and rest for at least 15 min with their legs uncrossed. Furthermore, the participant was asked to empty their bladder, not have caffeinated drinks before BP measurement, and should not talk or move during the measurements. The left arm was used to measure the BP and elbow was supported during the measurements. Three blood pressure measurements were performed and the mean of the second and third readings was calculated. The participant rested for 3 min between each measurement. Physical activity was defined based on the Global Physical Activity Questionnaire and classified based on the WHO recommendation into above and below the minimal level.^20^ An inverse relationship between household crowding and socioeconomic status has been previously established.^21^

### Data analysis

All analyses were performed using the STATA software version 17 (StataCorp LLC), accounting for the complex sampling design and sampling weights to produce representative estimates of the target population in Afghanistan. Descriptive statistics were used to describe the respondents’ sociodemographic, behavioral, and biochemical characteristics. Unadjusted (univariate) and adjusted (multivariate) multinomial logistic regression analyses were performed to estimate the associated factors of IFG and diabetes. Since the pregnancy hormones might influence the blood sugar level,^22^ 177 pregnant women were excluded in the final multivariable analysis to roll out gestational diabetes and its confounding effect; however, the prevalence of diabetes and IFG were reported for the whole population and excluding the pregnant women which showed no significant difference. Based on a comprehensive literature review, the relevant covariates including age, education, marital status, place of living, physical activity, serving fruit per day, tobacco and alcohol use, BMI, abdominal obesity, high cholesterol level, high blood pressure, and household members were included in the analysis. No multi-collinearity was detected except between BMI and abdominal circumferences; however, since the diabetogenic effect of these two variables is reported to interact,^23,24^ the interaction term was included in the final adjusted model. Missing data were excluded from the analysis, assuming complete randomness on the missing mechanism. Significance was set at *p* < 0.05.

### Ethics consideration

This was a secondary analysis of the Afghanistan WHO STEPwise approach to NCD risk factor surveillance in 2018 (STEPS 2018), an anonymized publicly-available dataset from Afghanistan. The Afghanistan STEPS 2018 protocol has been approved and guided by the Ministry of Public Health in Afghanistan. All participants were given a consent form before data collection that explained the study’s objectives, the confidentiality of the collected data, the fact that they could skip questions if they preferred not to, and that participation was entirely voluntary. Giving consent was regarded as taking part in the study. Given all ethical necessities were previously pursued and granted by involved parties, the secondary analysis of STEPS data requires no further ethical approval.

## Results

### Sample characteristics

Table 1 describes the distribution of participants’ sociodemographic characteristics and behavioral and biochemical profiles. The median age of participants was 35 years with an interquartile range of 25–48 years. More than half the participants were men (51.93%). The majority of participants had received no formal schooling (58.71%) and lived in households with four or more members (76.22%). Marital status varied with most participants being married (76.51%). Approximately 57.83% were living in urban areas. The majority of the sample reported consuming <3 servings of fruits and vegetables per day (84.2%). Approximately 73.05% of the participants had above the minimal recommended physical activity. Most of the participants had normal weight (49.46%), and a notable proportion had obesity (17.23%), with abdominal obesity observed in 60.67% of the participants. The majority were non-smokers (91.4%) and non-drinkers (99.5%). A significant proportion of participants had a high cholesterol level (18.4%) and high blood pressure (35.86%). An estimated 11.07% had diabetes, and 10.32% had IFG.

**Table 1.** The distribution of participants’ characteristics, behavioral, and biochemical profiles, Afghanistan STEPs 2018.

Variable	*n* (weighted%)
Median age (IQR)	35 (25–48)
Sex	
Male	1993 (51.93)
Female	1897 (48.07)
Education	
No schooling	2153 (58.71)
Primary/Secondary school	957 (24.6)
High/graduate school	780 (17.83)
Adult household member	
1–3	1396 (23.78)
⩾4	2494 (76.22)
Marital status	
Never married	596 (19.08)
Married	3132 (76.51)
Separated/divorced	161 (4.42)
Place of living	
Rural	1849 (42.17)
Urban	2041 (57.83)
Serving fruit and vegetable/day	
<3	3064 (84.2)
⩾3	731 (15.8)
Physical activity	
Below minimal recommendation	1034 (26.95)
Above minimal recommendation	2856 (73.05)
BMI (kg/m^2^)	
Underweight	258 (7.75)
Normal	1740 (49.46)
Overweight	1057 (25.57)
Obesity	634 (17.23)
Abdominal obesity	
No	1615 (39.33)
Yes	2275 (60.67)
Currently tobacco smoking	
No	3540 (91.4)
Yes	350 (8.6)
Ever alcohol consumption	
No	3846 (99.5)
Yes	44 (0.5)
High cholesterol	680 (18.4)
High blood pressure	1459 (35.86)
Diabetes	408 (11.07)
Diabetes (excluding pregnant women)	403 (11.67)
IFG	421 (10.32)
IFG (excluding pregnant women)	408 (10.57)

The numbers may not add up to the total number of 3890 due to missing data.

### Associated factors of diabetes and IFG among Afghan adults (18–70 years)

Tables 2 and 3 present the unadjusted and adjusted risk ratio for the association of participants’ sociodemographic, behavioral, and biochemical factors with diabetes profiles. The relative risk ratio (RRR) of developing IFG and diabetes increased with age per year, with an RRR of 1.03 (95% CI: 1.02–1.04) for IFG and 1.04 (95% CI: 1.02–1.05) for diabetes. In the adjusted model, the risk associated with age has slightly decreased for both IFG and diabetes, with an adjusted RRR (ARRR) of 1.02 for both conditions. Sex was not significantly associated with either IFG or diabetes. Individuals with no schooling had an elevated risk for diabetes with an RRR of 2.01 (95% CI: 1.13–3.59). However, in the adjusted model, this effect diminished compared to the unadjusted results, with no schooling having an ARRR of 1.59 for diabetes. No association between education and IFG was observed. Households with ⩾4 adult members also had a higher risk for IFG and diabetes than those with 1–3 members. Having ⩾4 adult household members significantly increased the risk of IFG (ARRR: 1.58) but not diabetes. However, this association was not significant in the unadjusted model, indicating that the effect was confounded by other variables. Regarding marital status, those who were separated/divorced had the highest risk for both IFG and diabetes compared to the never-married reference group. However, it has been largely attenuated in the adjusted model and became insignificant. Additionally, urban living was significantly associated with diabetes, with an RRR of 1.71 (95% CI: 1.12–2.62), and remained significant after adjustment (ARRR: 1.49). No significant association was observed between urban living and IFG.

**Table 2.** Unadjusted risk ratio (95% confidence interval) for the association between sociodemographic, behavioral, and biochemical factors and diabetes profile, Afghanistan STEPs 2018.

	IFG	Diabetes
Variables	RRR (95% CI)	RRR (95% CI)
Age (year)	1.03 (1.02, 1.04)	1.04 (1.02, 1.05)
Sex		
Male	Ref	Ref
Female	0.97 (0.58, 1.61)	1.11 (0.81, 1.51)
Education		
No schooling	1.31 (0.76, 2.26)	2.01 (1.13, 3.59)
Primary/Secondary school	0.77 (0.40, 1.46)	1.42 (0.73, 2.78)
High/graduate school	Ref	Ref
Adult household member		
1–3	Ref	Ref
⩾4	1.49 (0.98, 2.27)	1.59 (1.02, 2.48)
Marital status		
Never married	Ref	Ref
Married	2.75 (1.55, 4.87)	2.26 (1.27, 4.01)
Separated/divorced	5.07 (1.57, 16.43)	10.53 (4.35, 25.49)
Place of living		
Rural	Ref	Ref
Urban	1.39 (0.84, 2.28)	1.71 (1.12, 2.62)
Serving fruit/day		
<3	Ref	
⩾3	0.82 (0.51, 1.31)	0.61 (0.29, 1.26)
Physical activity		
Below minimal recommendation	Ref	Ref
Above minimal recommendation	0.67 (0.43, 1.04)	0.37 (0.23, 0.61)
BMI (kg/m^2^)		
Underweight	1.04 (0.42, 2.60)	0.83 (0.38, 1.84)
Normal	Ref	Ref
Overweight	1.59 (1.07, 2.35)	2.52 (1.56, 4.07)
Obesity	2.43 (1.57, 3.77)	4.73 (3.01, 7.43)
Abdominal obesity		
No	Ref	Ref
Yes	1.87 (1.25, 2.80)	3.43 (2.13, 5.52)
Currently tobacco smoking		
No	Ref	Ref
Yes	1.13 (0.54, 2.35)	1.23 (0.54, 2.81)
Ever alcohol consumption		
No	Ref	Ref
Yes	0.61 (0.13, 2.82)	0.29 (0.06, 1.47)
High cholesterol		
No	Ref	Ref
Yes	2.73 (1.57, 4.72)	6.08 (3.97, 9.30)
High blood pressure		
No	Ref	Ref
Yes	1.88 (1.33, 2.65)	4.71 (2.91, 7.64)

**Table 3.** Adjusted risk ratio (95% confidence interval) for the association between sociodemographic, behavioral, and biochemical factors and diabetes profile, Afghanistan STEPs 2018.

	IFG	Diabetes
Variables	ARRR (95%)	ARRR (95%)
Age (year)	1.02 (1.01, 1.03)	1.02 (1.00, 1.04)
Education		
No schooling	1.12 (0.66, 1.89)	1.59 (1.00, 2.51)
Primary/Secondary school	0.78 (0.45, 1.37)	1.66 (0.85, 3.26)
High/graduate school	Ref	
Marital status		
Never married	Ref	Ref
Married	1.69 (0.82, 3.46)	0.97 (0.49, 1.91)
Separated/divorced	1.81 (0.46, 7.03)	1.83 (0.77, 4.35)
Place of living		
Rural	Ref	Ref
Urban	1.37 (0.84, 2.24)	1.49 (1.00, 2.20)
Physical activity		
Below minimal recommendation	Ref	Ref
Above minimal recommendation	0.88 (0.57, 1.37)	0.53 (0.32, 0.88)
BMI# Abdominal obesity		
Underweight#No	1.03 (0.39, 2.71)	1.10 (0.35, 3.47)
Underweight#Yes	1.37 (0.17, 10.94)	1.24 (0.26, 5.88)
Normal#No	1.04 (0.56, 1.96)	1.47 (0.72, 2.99)
Normal#Yes	Ref	Ref
Overweight#No	0.80 (0.35, 1.83)	0.64 (0.27, 1.55)
Overweight#Yes	1.30 (0.84, 2.00)	2.12 (1.10, 4.09)
Obesity#No	0.78 (0.28, 2.22)	2.07 (0.62, 6.93)
Obesity#Yes	1.69 (0.87, 3.26)	2.54 (1.37, 4.70)
High cholesterol		
Yes	2.52 (1.55, 4.12)	4.12 (2.59, 6.56)
No	Ref	Ref
High blood pressure		
Yes	0.98 (0.64, 1.50)	1.82 (1.16, 2.86)
No	Ref	Ref
Adult household member		
1–3	Ref	Ref
⩾4	1.58 (1.05, 2.38)	1.21 (0.80, 1.83)

Physical activity above the minimal recommendation significantly reduced the risk of diabetes, with an RRR of 0.37 (95% CI: 0.23–0.61); it remained protective against diabetes in the adjusted model (ARRR: 0.53), albeit less than in the unadjusted model. No association between physical activity and IFG was identified. Individuals with obesity had the highest risk for both IFG (RRR: 2.43, 95% CI: 1.57–3.77) and diabetes (RRR: 4.73, 95% CI: 3.01–7.43). Abdominal obesity significantly increased the risk of both IFG and diabetes, with an RRR of 1.87 (95% CI: 1.25–2.80) and 3.43 (95% CI: 2.13–5.52) respectively. Tobacco smoking and alcohol consumption did not significantly alter the risk of IFG or diabetes. The significant interaction between BMI categories and abdominal obesity in the adjusted model was observed in the ‘overweight’ and ‘obesity’ categories. For individuals overweight with abdominal obesity, the risk for diabetes became significantly elevated, with an ARRR of 2.12 (95% CI: 1.10–4.09). However, the most pronounced effect was observed among individuals classified as having obesity with abdominal obesity with an ARRR of 2.54 (95% CI: 1.37–4.70).

Finally, high cholesterol levels and high blood pressure were significantly associated with an increased risk of both IFG and diabetes, with high cholesterol levels demonstrating the largest RRR of 6.08 (95% CI: 3.97–9.30) for diabetes. The effect of high cholesterol levels remained strong and significant for IFG and diabetes in the adjusted model (ARRR: 2.52 and 4.12, respectively). High blood pressure demonstrated a significantly increased risk for diabetes (ARRR: 1.82) but not for IFG.

## Discussion

To the best of our knowledge, this is the first study reporting on the prevalence of diabetes and IFG and elucidating the potential risk factors among a nationally representative sample of Afghan adults aged 18–69 years. Approximately 11.07% and 10.32% of Afghan adults had diabetes and IFG, respectively. Similar rates of diabetes were observed in previous regional studies with a diabetes prevalence of approximately 9.1%–13.2%.^5,6^ In a meta-analysis by Akhtar et al.,^3^ an estimated 12.13% of Afghan adults had diabetes. In their study, the higher diabetes prevalence could be attributed to heterogeneities in sample characteristics. Moreover, their study combined the data from five large provinces in Afghanistan in which rural areas were underrepresented. Additionally, the study sample was aged ⩾25 years and was not representative of younger adults.^3^ Nonetheless, the diabetes prevalence is higher than the global estimate of 10.5% in adults aged 20–79 years.^25^ This could be attributed to socioeconomic disparities and lifestyle variations, such as dietary patterns, physical activity levels, and obesity rates.^1^ Additionally, the present study suggested that older age, no schooling, living in urban areas, low physical activity, and high BMI combined with high abdominal circumference, hypertension, and hypercholesteremia are associated with a higher prevalence of diabetes in adjusted models, which was in line with those reported in previous studies.^26,27^

Age has been identified as a significant risk factor for diabetes, with older individuals being more susceptible to the condition.^26^ However, although age is a significant risk factor, it is not the sole determinant of developing diabetes,^28^ taking proactive measures, such as maintaining a healthy weight, being physically active, and managing other risk factors, can help delay or prevent the onset of diabetes, regardless of age.^29^ Regular screenings and discussions with healthcare professionals are also essential for the early detection, management, and prevention of diabetes-related complications. Additionally, studies have demonstrated that limited health literacy, referring to patients’ ability to understand and act on medical instructions, can negatively impact diabetes management and outcomes,^30^ consistent with the finding of our study indicating higher diabetes among those with no schooling. Therefore, addressing health literacy is crucial in empowering individuals to manage their diabetes effectively.^31^

In line with our study, the influence of living in urban areas on diabetes prevalence is supported by previous studies highlighting the impact of social and environmental factors on obesity and related chronic diseases.^27^ Growing urbanization in Afghanistan and accompanying lifestyle changes can lead to a transition in NCD risk factors. Those living in urban areas are reportedly more likely to have a sedentary lifestyle.^32^ Low physical activity is associated with a higher risk of obesity, which is associated with hypertension, dyslipidemia, and diabetes, emphasizing the importance of a balanced diet and regular physical activity for maintaining a healthy weight and preventing these conditions.^26,33^ Thus, the Afghan government should develop comprehensive national strategies to promote physical activity to prevent obesity and reduce the risk of diabetes. Moreover, investing in infrastructure to facilitate physical activity can increase population-wide physical activity levels. This includes creating safe and accessible spaces for exercise, such as parks, recreational facilities, and walking/cycling paths.^34^

An important finding in this study was the interaction between BMI and abdominal obesity. Although high BMI is a well-documented risk factor for diabetes,^35^ our results suggested the risk of diabetes is only higher in those with abdominal obesity across high BMI individuals. These findings highlight the importance of considering not only BMI but also the distribution of body fat, particularly abdominal fat when assessing the risk of diabetes. Individuals with a normal BMI but a high percentage of body fat are indicated to be at a greater risk of prediabetes or diabetes than individuals with lower body fat but a higher BMI.^36,37^ This implies that simply having a high BMI alone may not be sufficient to predict the risk of diabetes accurately. Some recent studies have even suggested waist-to-height ratio as a useful replacement for BMI and waist circumference in clinical screening.^38,39^

Another unique finding in the present study was reflecting on the prevalence of IFG. No previous study has reported on the IFG and its associated risk factors in Afghanistan. Although similar rates have been observed among the adult population in other countries such as Ethiopia,^40^ the results are inconsistent across countries, which might be because of the variation in diagnostic criteria and methodology,^28^ demographic and population characteristics,^41^ and lifestyle factors and obesity^42^ across different studies. However, the prevalence of IFG for Afghan adults in this study may be higher than the global average for low-income countries (5.8%). Considering the similarities in the underlying causes of risk factors for IFG and diabetes, targeted health promotion and education programs must be implemented to raise awareness regarding risk factors, prevention, and management of diabetes and IFG. This can include campaigns promoting healthy lifestyles, regular physical activity, and balanced diets.^41^

The study had several notable strengths. First, this is one of the first reports providing nationally representative estimates for diabetes and IFG, including rural and urban residents. This enhances the generalizability of the findings. Second, the study reports on diabetes and IFG, providing a comprehensive understanding of their prevalence and associated risk, thus allowing for informed decision-making and developing targeted strategies in Afghanistan. However, the study had some limitations. First, the data on the study variables, such as fruit and vegetable consumption, tobacco use, alcohol use, and physical activity, were self-reported, introducing the possibility of information bias and distorting the association between these variables and diabetes. Second, the cross-sectional study design of this study limits our ability to establish causal relationships. Third, the lack of data on the chronicity and type of treatment prohibited the distinguishment between types of diabetes. Moreover, Afghanistan STEPS 2018 only measured and reported total cholesterol; hence, it is not possible to report on subcomponents, including LDL- and HDL-cholesterol. Acknowledging the potential influence of reverse causality is important, as participants who already had IFG or diabetes before the study might have modified their diet and lifestyle. Thus, large-scale cohort studies should be implemented in Afghanistan.

## Conclusions

This study elucidates the prevalence of diabetes and IFG among Afghan adults aged 18–69 years. Higher prevalence of these conditions as compared to the global average requires tailored interventions that target the population at risk, such as the older population, those with no schooling, urban residents, those with cardiometabolic risk factors, such as low physical activity, high BMI, abdominal obesity, hypertension, and hypercholesteremia. Despite the continuous efforts of the Afghanistan Ministry of Public Health, NCD-related health service delivery remains limited across the country, and the NCD rate is growing rapidly in Afghanistan.^4^ Addressing limited health literacy is particularly crucial for effective diabetes management. National strategies should also promote and encourage physical activity by investing in infrastructures, such as safe and accessible spaces for both men and women to exercise.

## Supplemental Material

sj-doc-1-smo-10.1177_20503121241238147 – Supplemental material for Prevalence and correlates of diabetes and impaired fasting glucose among adults in Afghanistan: Insights from a national survey Supplemental material, sj-doc-1-smo-10.1177_20503121241238147 for Prevalence and correlates of diabetes and impaired fasting glucose among adults in Afghanistan: Insights from a national survey by Omid Dadras, Anita Nyaboke Ongosi and Chia-Wen Wang in SAGE Open Medicine
