# Supplementary material for: Prevalence and correlates of diabetes and impaired fasting glucose among adults in Afghanistan: Insights from a national survey
Source: SAGE Open Med. 2024 Mar 20;12:20503121241238147. doi: 10.1177/20503121241238147 (PMC10956149; doi:10.1177/20503121241238147)
Supplement: sj-doc-1-smo-10.1177_20503121241238147 – Supplemental material for Prevalence and correlates of diabetes and impaired fasting glucose among adults in Afghanistan: Insights from a national survey [file sj-doc-1-smo-10.1177_20503121241238147.doc]

**WHO STEPS Instrument**

**(Core and Expanded)**


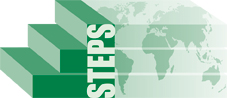


The WHO STEPwise approach to noncommunicable disease risk factor surveillance (STEPS)


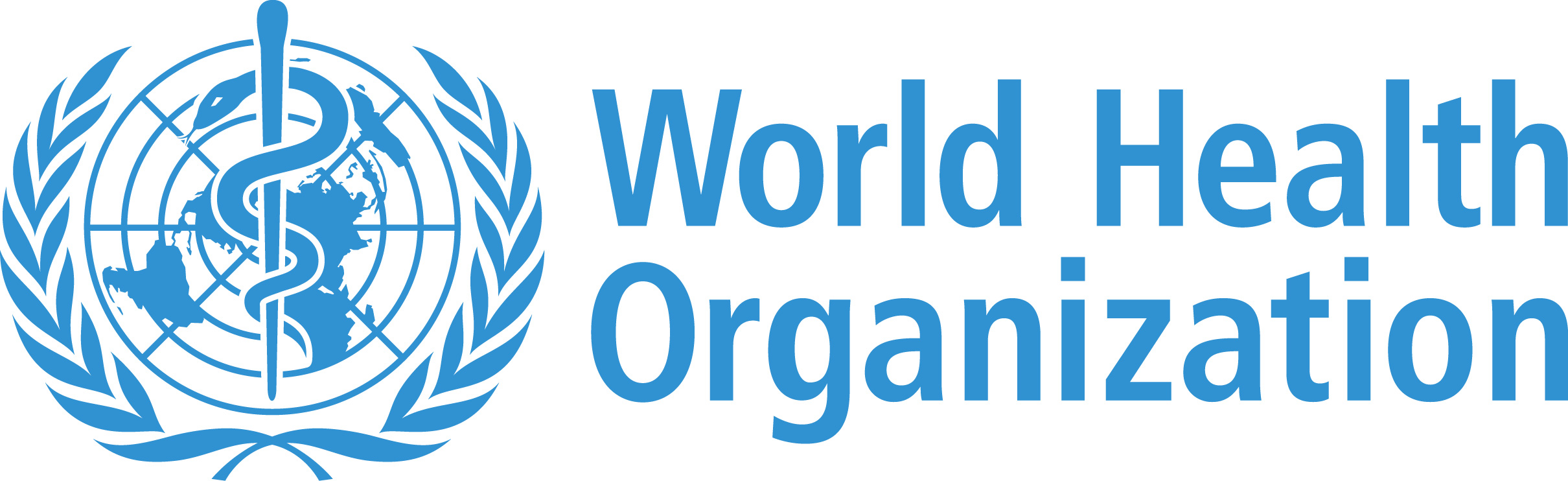
World Health Organization

20 Avenue Appia, 1211 Geneva 27, Switzerland

*For further information:* [www.who.int/chp/steps](http://www.who.int/chp/steps)

# STEPS Instrument

#### Overview

| Introduction | This is the generic STEPS Instrument which sites/countries will use to develop their tailored instrument. It contains the:   - CORE items (unshaded boxes) - EXPANDED items (shaded boxes). |
| --- | --- |

| Core Items | The Core items for each section ask questions required to calculate basic variables. For example:   - current daily smokers - mean BMI. |
| --- | --- |
|  | **Note:** All the core questions should be asked, removing core questions will impact the analysis. |

| Expanded items | The Expanded items for each section ask more detailed information. Examples include:   - use of smokeless tobacco - sedentary behaviour. |
| --- | --- |

| Guide to the columns | The table below is a brief guide to each of the columns in the Instrument. |
| --- | --- |

| Column | Description | Site Tailoring |
| --- | --- | --- |
| Question | Each question is to be read to the participants | - Select sections to use. - Add expanded and optional questions as desired. |
| Response | This column lists the available response options which the interviewer will be circling or filling in the text boxes. The skip instructions are shown on the right hand side of the responses and should be carefully followed during interviews. | - Add site specific responses for demographic responses (e.g. C6). - Change skip question identifiers where necessary. |
| Code | The column is designed to match data from the instrument into the data entry tool, data analysis syntax, data book, and fact sheet. | This should never be changed or removed. The code is used as a general identifier for the data entry and analysis. |


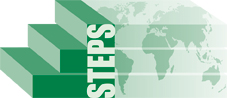
WHO STEPS Instrument

for Noncommunicable Disease
Risk Factor Surveillance

<insert country/site name>

| **Survey Information** |
| --- |

| Location and Date | | Response | | Code | |
| --- | --- | --- | --- | --- | --- |
| Cluster/Centre/Village ID | └─┴─┴─┴─┴─┴─┘ | | I1 | |  |
| Cluster/Centre/Village name |  | | I2 | |  |
| Interviewer ID | └─┴─┴─┘ | | I3 | |  |
| Date of completion of the instrument | └─┴─┘ └─┴─┘ └─┴─┴─┴─┘ dd mm year | | I4 | |  |

| Consent, Interview Language and Name | Response | | Code |
| --- | --- | --- | --- |
| Consent has been read and obtained | Yes | 1 | I5 |
|  | No | 2 **If NO, END** |  |
| Interview Language *[Insert Language]* | English | 1 | I6 |
|  | Dari | 2 |  |
|  | Pashto | 3 |  |
| Time of interview  (24 hour clock) | └─┴─┘: └─┴─┘  hrs mins | | I7 |
| Family Surname |  | | I8 |
| First Name |  | | I9 |
| **Additional Information that may be helpful** | | | |
| Contact phone number where possible |  | | I10 |

| **Step 1 Demographic Information** |
| --- |

| **CORE: Demographic Information** | | | | |
| --- | --- | --- | --- | --- |
| **Question** | **Response** | | | **Code** |
| Sex (*Record Male / Female as observed)* | Male | | 1 | C1 |
|  | Female | | 2 |  |
| What is your date of birth?  *Don't Know 77 77 7777* | └─┴─┘ └─┴─┘ └─┴─┴─┴─┘ *If known, Go to C4*  dd mm year | | | C2 |
| How old are you? | Years | | └─┴─┘ | C3 |
| In total, how many years have you spent at school and in full-time study (excluding pre-school)? | Years | └─┴─┘ | | C4 |

| **EXPANDED: Demographic Information** | | | |
| --- | --- | --- | --- |
| What is the **highest level of education** you have completed?  *[INSERT COUNTRY-SPECIFIC CATEGORIES]* | No formal schooling | 1 | C5 |
|  | Less than primary school | 2 |  |
|  | Primary school completed | 3 |  |
|  | Secondary school completed | 4 |  |
|  | High school completed | 5 |  |
|  | College/University completed | 6 |  |
|  | Post graduate degree | 7 |  |
|  | Refused | 88 |  |
| What is your **marital status**? | Never married | 1 | C7 |
|  | Currently married | 2 |  |
|  | Separated | 3 |  |
|  | Divorced | 4 |  |
|  | Widowed | 5 |  |
|  | Refused | 88 |  |
| Which of the following best describes your **main** **work** status over the past 12 months?  *[INSERT COUNTRY-SPECIFIC CATEGORIES]*  (*USE SHOWCARD)* | Government employee | 1 | C8 |
|  | Non-government employee | 2 |  |
|  | Self-employed | 3 |  |
|  | Non-paid | 4 |  |
|  | Student | 5 |  |
|  | Homemaker | 6 |  |
|  | Retired | 7 |  |
|  | Unemployed (able to work) | 8 |  |
|  | Unemployed (unable to work) | 9 |  |
|  | Refused | 88 |  |
| How many people older than 18 years, including yourself, live in your household? | Number of people | └─┴─┘ | C9 |

| **EXPANDED: Demographic Information,** Continued | | | | |
| --- | --- | --- | --- | --- |
| **Question** | **Response** | | | **Code** |
| Taking **the past year**, can you tell me what the average earnings of the household have been?  *(RECORD ONLY ONE, NOT ALL 3)* | Per week | └─┴─┴─┴─┴─┴─┴─┘ *Go to T1* | | C10a |
|  | OR per month | └─┴─┴─┴─┴─┴─┴─┘  *Go to T1* | | C10b |
|  | OR per year | └─┴─┴─┴─┴─┴─┴─┘ *Go to T1* | | C10c |
|  | Refused | 88 | | C10d |
| If you don’t know the amount, can you give an **estimate** of the annual household income if I read some options to you? Is it  *[INSERT QUINTILE VALUES IN LOCAL CURRENCY]*  *(READ OPTIONS)* | ≤ Quintile (Q) 1 | | 1 | C11 |
|  | More than Q 1, ≤ Q 2 | | 2 |  |
|  | More than Q 2, ≤ Q 3 | | 3 |  |
|  | More than Q 3, ≤ Q 4 | | 4 |  |
|  | More than Q 4 | | 5 |  |
|  | Don't Know | | 77 |  |
|  | Refused | | 88 |  |

| **Step 1 Behavioural Measurements** |
| --- |

| **CORE: Tobacco Use** | | | |
| --- | --- | --- | --- |
| Now I am going to ask you some questions about tobacco use. | | | |
| **Question** | **Response** | | **Code** |
| Do you **currently** smoke any **tobacco** products, such as cigarettes, cigars or pipes?  *(USE SHOWCARD)* | Yes | 1 | T1 |
|  | No | 2 *If No, go to T8* |  |
| Do you currently smoke tobacco products **daily**? | Yes | 1 | T2 |
|  | No | 2 |  |
| How old were you when you **first started** smoking? | Age (years) | **└─┴─┘** *If Known, go to T5a/T5aw* | T3 |
|  | Don’t know 77 |  |  |
| Do you remember how long ago it was?  *(RECORD ONLY 1, NOT ALL 3)*  *Don’t know 77* | In Years | **└─┴─┘** *If Known, go to T5a/T5aw* | T4a |
|  | OR in Months | **└─┴─┘** *If Known, go to T5a/T5aw* | T4b |
|  | OR in Weeks | **└─┴─┘** | T4c |
| On average, **how many** of the following products do you smoke **each day/week?**  *(IF LESS THAN DAILY, RECORD WEEKLY)*  *(RECORD FOR EACH TYPE, USE SHOWCARD)*  *Don’t Know 7777* | DAILY**↓** WEEKLY↓ | | |
|  | Manufactured cigarettes | └─┴─┴─┴**─┘**└─┴─┴─┴**─┘** | T5a/T5aw |
|  | Hand-rolled cigarettes | └─┴─┴─┴**─┘**└─┴─┴─┴**─┘** | T5b/T5bw |
|  | Pipes full of tobacco | └─┴─┴─┴**─┘**└─┴─┴─┴**─┘** | T5c/T5cw |
|  | Cigars, cheroots, cigarillos | └─┴─┴─┴**─┘**└─┴─┴─┴**─┘** | T5d/T5dw |
|  | Number of Shisha sessions | └─┴─┴─┴**─┘**└─┴─┴─┴**─┘** | T5e/T5ew |
|  | Other | └─┴─┴─┴**─┘**└─┴─┴─┴**─┘** *If Other, go to T5other, else go to T6* | T5f/T5fw |
|  | Other (please specify): | └─┴─┴─┴─┴─┴─┘ | T5other/  T5otherw |
| During the past 12 months, have you tried to **stop smoking**? | Yes | 1 | T6 |
|  | No | 2 |  |
| During any visit to a doctor or other health worker in the past 12 months, were you advised to quit smoking tobacco? | Yes | 1 *If T2=Yes, go to T12; if T2=No, go* *to T9* | T7 |
|  | No | 2 *If T2=Yes, go to T12; if T2=No, go to T9* |  |
|  | No visit during the past 12 months | 3 *If T2=Yes, go to T12; if T2=No, go to T9* |  |
| In the past, did you **ever** **smoke** any tobacco products?  *(USE SHOWCARD)* | Yes | 1 | T8 |
|  | No | 2 *If No, go to T12* |  |
| In the past, did you **ever** smoke **daily**? | Yes | 1 *If T1=Yes, go to T12, else go to T10* | T9 |
|  | No | 2 *If T1=Yes, go to T12, else go to T10* |  |

| **EXPANDED: Tobacco Use** | | | |
| --- | --- | --- | --- |
| **Question** | **Response** | | **Code** |
| How old were you when you **stopped** smoking? | Age (years) | **└─┴─┘** *If Known, go to T12* | T10 |
|  | Don’t Know 77 |  |  |
| How **long ago** did you stop smoking?  *(RECORD ONLY 1, NOT ALL 3)*  *Don’t Know 77* | Years ago | **└─┴─┘** *If Known, go to T12* | T11a |
|  | OR Months ago | **└─┴─┘** *If Known, go to T12* | T11b |
|  | OR Weeks ago | **└─┴─┘** | T11c |
| Do you **currently use** any **smokeless tobacco** products such as *[snuff, chewing tobacco, betel]*? *(USE SHOWCARD)* | Yes | 1 | T12 |
|  | No | 2 *If No, go to T15* |  |
| Do you **currently use** **smokeless tobacco** products **daily?** | Yes | 1 | T13 |
|  | No | 2 *If No, go to T14aw* |  |
| On average, how many **times a day/week** do you use ….  *(IF LESS THAN DAILY, RECORD WEEKLY)*  *(RECORD FOR EACH TYPE, USE SHOWCARD)*  *Don’t Know 7777* | DAILY**↓** WEEKLY↓ | | |
|  | Snuff, by mouth | └─┴─┴─┴**─┘**└─┴─┴─┴**─┘** | T14a/  T14aw |
|  | Snuff, by nose | └─┴─┴─┴**─┘**└─┴─┴─┴**─┘** | T14b/  T14bw |
|  | Chewing tobacco | └─┴─┴─┴**─┘**└─┴─┴─┴**─┘** | T14c/  T14cw |
|  | Betel, quid | └─┴─┴─┴**─┘**└─┴─┴─┴**─┘** | T14d/  T14dw |
|  | Other | └─┴─┴─┴**─┘**└─┴─┴─┴**─┘** *If Other, go to T14other, if T13=No, go to T16, else go to T17* | T14e/  T14ew |
|  | Other (please specify): | └─┴─┴─┴─┴─┴─┘  *If T13=No, go to T16, else go to T17* | T14other/  T14otherw |
| In the **past**, did you **ever use** smokeless tobacco products such as *[snuff, chewing tobacco, or betel]*? | Yes | 1 | T15 |
|  | No | 2 *If No, go to T17* |  |
| In the **past**, did you **ever use** smokeless tobacco products such as *[snuff, chewing tobacco, or betel]* **daily**? | Yes | 1 | T16 |
|  | No | 2 |  |
| During the past 30 days, did someone smoke **in your home**? | Yes | 1 | T17 |
|  | No | 2 |  |
| During the past 30 days, did someone smoke in closed areas **in your workplace** (in the building, in a work area or a specific office)? | Yes | 1 | T18 |
|  | No | 2 |  |
|  | Don't work in a closed area | 3 |  |

| **CORE: Alcohol Consumption** | | | |
| --- | --- | --- | --- |
| The next questions ask about the consumption of alcohol. | | | |
| **Question** | **Response** | | **Code** |
| Have you **ever** consumed any alcohol such as beer, wine, spirits or *[add other local examples]*?  *(USE SHOWCARD OR SHOW EXAMPLES)* | Yes | 1 | A1 |
|  | No | 2  *If No, go to A16* |  |
| Have you consumed any alcohol within the **past 12 months**? | Yes | 1  *If Yes, go to A4* | A2 |
|  | No | 2 |  |
| Have you stopped drinking due to health reasons, such as a negative impact on your health or on the advice of your doctor or other health worker? | Yes | 1 *If Yes, go to A16* | A3 |
|  | No | 2 *If No, go to A16* |  |
| During the past 12 months, **how frequently** have you had at least one standard alcoholic drink?  *(READ RESPONSES, USE SHOWCARD)* | Daily | 1 | A4 |
|  | 5-6 days per week | 2 |  |
|  | 3-4 days per week | 3 |  |
|  | 1-2 days per week | 4 |  |
|  | 1-3 days per month | 5 |  |
|  | Less than once a month | 6 |  |
| Have you consumed any alcohol within the **past 30 days**? | Yes | 1 | A5 |
|  | No | 2 *If No, go to A13* |  |
| During the past 30 days, on how many **occasions** did you have at least one standard alcoholic drink? | Number  Don't know 77 | └─┴─┘ | A6 |
| During the past 30 days, when you drank alcohol, how many **standard** **drinks on average** did you have during one drinking occasion?  *(USE SHOWCARD)* | Number  Don't know 77 | └─┴─┘ | A7 |
| During the past 30 days, what was the **largest number** of standard drinks you had on a single occasion, counting all types of alcoholic drinks together? | Largest number  Don't Know 77 | └─┴─┘ | A8 |
| During the past 30 days, how many times did you have  **six or more** standard drinks in a single drinking occasion? | Number of times Don't Know 77 | └─┴─┘ | A9 |
| During each of the **past 7 days**, how many standard drinks did you have each day?  *(USE SHOWCARD)*  *Don't Know 77* | Monday | └─┴─┘ | A10a |
|  | Tuesday | └─┴─┘ | A10b |
|  | Wednesday | └─┴─┘ | A10c |
|  | Thursday | └─┴─┘ | A10d |
|  | Friday | └─┴─┘ | A10e |
|  | Saturday | └─┴─┘ | A10f |
|  | Sunday | └─┴─┘ | A10g |

| **CORE: Alcohol Consumption, continued** | | | |
| --- | --- | --- | --- |
| I have just asked you about your consumption of alcohol during the past 7 days. The questions were about alcohol in general, while the next questions refer to your consumption of homebrewed alcohol, alcohol brought over the border/from another country, any alcohol not intended for drinking or other untaxed alcohol. Please only think about these types of alcohol when answering the next questions. | | | |
| **Question** | **Response** | | **Code** |
| During the **past 7 days**, did you consume any **homebrewed** alcohol, any alcohol **brought over the border/from another country**, any alcohol **not intended for drinking** or other **untaxed** alcohol?  *[AMEND ACCORDING TO LOCAL CONTEXT]*  *(USE SHOWCARD)* | Yes | 1 | A11 |
|  | No | 2 *If No, go to A13* |  |
| On average, **how many standard drinks** of the following did you consume **during the past 7 days**?  *[INSERT COUNTRY-SPECIFIC EXAMPLES]*  *(USE SHOWCARD)*  *Don't Know 77* | Homebrewed spirits, e.g. moonshine | **└─┴─┘** | A12a |
|  | Homebrewed beer or wine, e.g. beer, palm or fruit wine | **└─┴─┘** | A12b |
|  | Alcohol brought over the border/from another country | **└─┴─┘** | A12c |
|  | Alcohol not intended for drinking, e.g. alcohol-based medicines, perfumes, after shaves | **└─┴─┘** | A12d |
|  | Other untaxed alcohol in the country | **└─┴─┘** | A12e |

| **CORE: Diet** | | | | | | |  |
| --- | --- | --- | --- | --- | --- | --- | --- |
| The next questions ask about the fruits and vegetables that you usually eat. I have a nutrition card here that shows you some examples of local fruits and vegetables. Each picture represents the size of a serving. As you answer these questions please think of a typical week in the last year. | | | | | | |  |
| **Question** | **Response** | | | | **Code** | |  |
| In a typical week, on how many days do you **eat fruit**?  *(USE SHOWCARD)* | Number of days Don't Know 77 | | └─┴─┘ If Zero days, go to D3 | | D1 | |  |
| How many **servings** of fruit do you eat on **one** of those days? (*USE SHOWCARD)* | Number of servings  Don't Know 77 | | └─┴─┘ | | D2 | |  |
| In a typical week, on how many days do you **eat vegetables**? *(USE SHOWCARD)* | Number of days Don't Know 77 | | └─┴─┘ If Zero days, go to D5 | | D3 | |  |
| How many **servings** of vegetables do you eat on one of those days? *(USE SHOWCARD)* | Number of servings  Don’t know 77 | | └─┴─┘ | | D4 | |  |
| **Dietary salt** | | | | | | | |
| With the next questions, we would like to learn more about salt in your diet. Dietary salt includes ordinary table salt, unrefined salt such as sea salt, iodized salt, salty stock cubes and powders, and salty sauces such as soya sauce or fish sauce (see showcard). The following questions are on adding salt to the food right before you eat it, on how food is prepared in your home, on eating processed foods that are high in salt such as [insert country specific examples], and questions on controlling your salt intake. Please answer the questions even if you consider yourself to eat a diet low in salt. | | | | | | | |
| How often do you **add salt or a salty sauce such as soya sauce** to your food right before you eat it or as you are eating it?  *(SELECT ONLY ONE)*  *(USE SHOWCARD)* | | Always | | 1 | | D5 | |
|  |  | Often | | 2 | |  |  |
|  |  | Sometimes | | 3 | |  |  |
|  |  | Rarely | | 4 | |  |  |
|  |  | Never | | 5 | |  |  |
|  |  | Don't know | | 77 | |  |  |
| How often is **salt, salty seasoning or a salty sauce added** in cooking or preparing foods in your household? | | Always | | 1 | | D6 | |
|  |  | Often | | 2 | |  |  |
|  |  | Sometimes | | 3 | |  |  |
|  |  | Rarely | | 4 | |  |  |
|  |  | Never | | 5 | |  |  |
|  |  | Don't know | | 77 | |  |  |
| How often do you eat **processed food high in salt**? By processed food high in salt, I mean foods that have been altered from their natural state, such as packaged salty snacks, canned salty food including pickles and preserves, salty food prepared at a fast food restaurant, cheese, bacon and processed meat *[add country specific examples].*  *[INSERT EXAMPLES]*  *(USE SHOWCARD)* | | Always | | 1 | | D7 | |
|  |  | Often | | 2 | |  |  |
|  |  | Sometimes | | 3 | |  |  |
|  |  | Rarely | | 4 | |  |  |
|  |  | Never | | 5 | |  |  |
|  |  | Don't know | | 77 | |  |  |
| **How much salt or salty sauce** do you think you consume? | | Far too much | | 1 | | D8 | |
|  |  | Too much | | 2 | |  |  |
|  |  | Just the right amount | | 3 | |  |  |
|  |  | Too little | | 4 | |  |  |
|  |  | Far too little | | 5 | |  |  |
|  |  | Don't know | | 77 | |  |  |

| **EXPANDED: Diet** | | | | | | | | | | | |
| --- | --- | --- | --- | --- | --- | --- | --- | --- | --- | --- | --- |
| **Question** | | | | **Response** | | | | | | | **Code** |
| How important to you is **lowering the salt** in your diet? | | Very important | | | | | 1 | D9 | | | |
|  |  | Somewhat important | | | | | 2 |  |  |  |  |
|  |  | Not at all important | | | | | 3 |  |  |  |  |
|  |  | Don't know | | | | | 77 |  |  |  |  |
| Do you think that too much salt or salty sauce in your diet could cause a **health problem**? | | Yes | | | | | 1 | D10 | | | |
|  |  | No | | | | | 2 |  |  |  |  |
|  |  | Don't know | | | | | 77 |  |  |  |  |
| Do you do any of the following on a regular basis to **control your salt intake**?  *(RECORD FOR EACH)* | | | | | | | | | | | |
| Limit consumption of processed foods | | | Yes | | | 1 | | | | D11a | |
|  |  |  | No | | | 2 | | | |  |  |
| Look at the salt or sodium content on food labels | | | Yes | | | 1 | | | | D11b | |
|  |  |  | No | | | 2 | | | |  |  |
| Buy low salt/sodium alternatives | | | Yes | | | 1 | | | | D11c | |
|  |  |  | No | | | 2 | | | |  |  |
| Use spices other than salt when cooking | | | Yes | | | 1 | | | | D11d | |
|  |  |  | No | | | 2 | | | |  |  |
| Avoid eating foods prepared outside of a home | | | Yes | | | 1 | | | | D11e | |
|  |  |  | No | | | 2 | | | |  |  |
| Do other things specifically to control your salt intake | | | Yes | | | 1  *If Yes, go to D11other* | | | | D11f | |
|  |  |  | No | | | 2 | | | |  |  |
| Other (please specify) | | | └─┴─┴─┴─┴─┴─┴─┘ | | | | | | | D11other | |
| The next questions ask about the oil or fat that is most often used for meal preparation in your household, and about meals that you eat outside a home. | | | | | | | | | | | |
| What type of **oil or fat is most often** used for meal preparation in your household?  *(USE SHOWCARD)*  *(SELECT ONLY ONE)* | Vegetable oil | | | | 1 | | | | D12 | | |
|  | Lard or suet | | | | 2 | | | |  |  |  |
|  | Butter or ghee | | | | 3 | | | |  |  |  |
|  | Margarine | | | | 4 | | | |  |  |  |
|  | Other | | | | 5  *If Other, go to D12 other* | | | |  |  |  |
|  | None in particular | | | | 6 | | | |  |  |  |
|  | None used | | | | 7 | | | |  |  |  |
|  | Don’t know | | | | 77 | | | |  |  |  |
|  | Other | | | | └─┴─┴─┴─┴─┴─┴─┘ | | | | D12other | | |
| On average, how many meals per week do you eat that were not prepared at a home? By meal, I mean breakfast, lunch and dinner. | Number  Don’t know 77 | | | | └─┴─┘ | | | | D13 | | |

| **CORE: Physical Activity** | | | |
| --- | --- | --- | --- |
| Next I am going to ask you about the time you spend doing different types of physical activity in a typical week. Please answer these questions even if you do not consider yourself to be a physically active person.  Think first about the time you spend doing work. Think of work as the things that you have to do such as paid or unpaid work, study/training, household chores, harvesting food/crops, fishing or hunting for food, seeking employment. *[Insert other examples if needed].* In answering the following questions 'vigorous-intensity activities' are activities that require hard physical effort and cause large increases in breathing or heart rate, 'moderate-intensity activities' are activities that require moderate physical effort and cause small increases in breathing or heart rate. | | | |
| **Question** | **Response** | | **Code** |
| **Work** | | | |
| Does your work involve vigorous-intensity activity that causes large increases in breathing or heart rate like *[carrying or lifting* *heavy loads, digging or construction work]*  for at least 10 minutes continuously?  *[INSERT EXAMPLES] (USE SHOWCARD)* | Yes | 1 | P1 |
|  | No | 2  *If No, go to P 4* |  |
| In a typical week, on how many days do you do vigorous-intensity activities as part of your work? | Number of days | └─┘ | P2 |
| How much time do you spend doing vigorous-intensity activities at work on a typical day? | Hours : minutes | └─┴─┘: └─┴─┘  hrs mins | P3 (a-b) |
| Does your work involve moderate-intensity activity, that causes small increases in breathing or heart rate such as brisk walking *[or carrying light loads]* for at least 10 minutes continuously?  *[INSERT EXAMPLES] (USE SHOWCARD)* | Yes | 1 | P4 |
|  | No | 2 *If No, go to P 7* |  |
| In a typical week, on how many days do you do moderate-intensity activities as part of your work? | Number of days | └─┘ | P5 |
| How much time do you spend doing moderate-intensity activities at work on a typical day? | Hours : minutes | └─┴─┘: └─┴─┘  hrs mins | P6 (a-b) |
| **Travel to and from places** | | | |
| The next questions exclude the physical activities at work that you have already mentioned.  Now I would like to ask you about the usual way you travel to and from places. For example to work, for shopping, to market, to place of worship. *[Insert other examples if needed]* | | | |
| Do you walk or use a bicycle *(pedal cycle)* for at least 10 minutes continuously to get to and from places? | Yes | 1 | P7 |
|  | No | 2  *If No, go to P 10* |  |
| In a typical week, on how many days do you walk or bicycle for at least 10 minutes continuously to get to and from places? | Number of days | └─┘ | P8 |
| How much time do you spend walking or bicycling for travel on a typical day? | Hours : minutes | └─┴─┘: └─┴─┘  hrs mins | P9 (a-b) |

| **CORE: Physical Activity, Continued** | | | |
| --- | --- | --- | --- |
| **Question** | **Response** | | **Code** |
| **Recreational activities** | | | |
| The next questions exclude the work and transport activities that you have already mentioned.  Now I would like to ask you about sports, fitness and recreational activities (leisure), *[Insert relevant terms]*. | | | |
| Do you do any vigorous-intensity sports, fitness or recreational *(leisure)* activities that cause large increases in breathing or heart rate like *[running or football]*  for at least 10 minutes continuously?  *[INSERT EXAMPLES] (USE SHOWCARD)* | Yes | 1 | P10 |
|  | No | 2  *If No, go to P 13* |  |
| In a typical week, on how many days do you do vigorous-intensity sports, fitness or recreational *(leisure)* activities? | Number of days | └─┘ | P11 |
| How much time do you spend doing vigorous-intensity sports, fitness or recreational activities on a typical day? | Hours : minutes | └─┴─┘: └─┴─┘  hrs mins | P12  (a-b) |
| Do you do any moderate-intensity sports, fitness or recreational *(leisure)* activities that cause a small increase in breathing or heart rate such as brisk walking*, [cycling, swimming, volleyball]* for at least 10 minutes continuously?  *[INSERT EXAMPLES] (USE SHOWCARD)* | Yes | 1 | P13 |
|  | No | 2  *If No, go to P16* |  |
| In a typical week, on how many days do you do moderate-intensity sports, fitness or recreational *(leisure)* activities? | Number of days | └─┘ | P14 |
| How much time do you spend doing moderate-intensity sports, fitness or recreational *(leisure)* activities on a typical day? | Hours : minutes | └─┴─┘: └─┴─┘  hrs mins | P15 (a-b) |

| **EXPANDED: Physical Activity** | | | |
| --- | --- | --- | --- |
| **Sedentary behaviour** | | | |
| The following question is about sitting or reclining at work, at home, getting to and from places, or with friends including time spent sitting at a desk, sitting with friends, traveling in car, bus, train, reading, playing cards or watching television, but do not include time spent sleeping.  *[INSERT EXAMPLES] (USE SHOWCARD)* | | | |
| How much time do you usually spend sitting or reclining on a typical day? | Hours : minutes | └─┴─┘: └─┴─┘  hrs mins | P16  (a-b) |

| **CORE: History of Raised Blood Pressure** | | | |
| --- | --- | --- | --- |
| **Question** | **Response** | | **Code** |
| Have you ever had your blood pressure measured by a doctor or other health worker? | Yes | 1 | H1 |
|  | No | 2 *If No, go to H6* |  |
| Have you ever been told by a doctor or other health worker that you have raised blood pressure or hypertension? | Yes | 1 | H2a |
|  | No | 2  *If No, go to H6* |  |
| Have you been told in the past 12 months? | Yes | 1 | H2b |
|  | No | 2 |  |
| In the past two weeks, have you taken any drugs (medication) for raised blood pressure prescribed by a doctor or other health worker? | Yes | 1 | H3 |
|  | No | 2 |  |
| Have you ever seen a traditional healer for raised blood pressure or hypertension? | Yes | 1 | H4 |
|  | No | 2 |  |
| Are you currently taking any herbal or traditional remedy for your raised blood pressure? | Yes | 1 | H5 |
|  | No | 2 |  |

| **CORE: History of Diabetes** | | | |
| --- | --- | --- | --- |
| Have you ever had your blood sugar measured by a doctor or other health worker? | Yes | 1 | H6 |
|  | No | 2 *If No, go to H12* |  |
| Have you ever been told by a doctor or other health worker that you have raised blood sugar or diabetes? | Yes | 1 | H7a |
|  | No | 2  *If No, go to H12* |  |
| Have you been told in the past 12 months? | Yes | 1 | H7b |
|  | No | 2 |  |
| In the past two weeks, have you taken any drugs (medication) for diabetes prescribed by a doctor or other health worker? | Yes | 1 | H8 |
|  | No | 2 |  |
| Are you currently taking insulin for diabetes prescribed by a doctor or other health worker? | Yes | 1 | H9 |
|  | No | 2 |  |
| Have you ever seen a traditional healer for diabetes or raised blood sugar? | Yes | 1 | H10 |
|  | No | 2 |  |
| Are you currently taking any herbal or traditional remedy for your diabetes? | Yes | 1 | H11 |
|  | No | 2 |  |

| **CORE: History of Raised Total Cholesterol** | | | |
| --- | --- | --- | --- |
| **Question** | **Response** | | **Code** |
| Have you ever had your cholesterol (fat levels in your blood) measured by a doctor or other health worker? | Yes | 1 | H12 |
|  | No | 2 *If No, go to H17* |  |
| Have you ever been told by a doctor or other health worker that you have raised cholesterol? | Yes | 1 | H13a |
|  | No | 2 *If No, go to H17* |  |
| Have you been told in the past 12 months? | Yes | 1 | H13b |
|  | No | 2 |  |
| In the past two weeks, have you taken any oral treatment (medication) for raised total cholesterol prescribed by a doctor or other health worker? | Yes | 1 | H14 |
|  | No | 2 |  |
| Have you ever seen a traditional healer for raised cholesterol? | Yes | 1 | H15 |
|  | No | 2 |  |
| Are you currently taking any herbal or traditional remedy for your raised cholesterol? | Yes | 1 | H16 |
|  | No | 2 |  |

| **CORE: History of Cardiovascular Diseases** | | | |
| --- | --- | --- | --- |
| Have you ever had a heart attack or chest pain from heart disease (angina) or a stroke (cerebrovascular accident or incident)? | Yes | 1 | H17 |
|  | No | 2 |  |
| Are you currently taking aspirin regularly to prevent or treat heart disease? | Yes | 1 | H18 |
|  | No | 2 |  |
| Are you currently taking statins (Lovastatin/Simvastatin/Atorvastatin or any other statin) regularly to prevent or treat heart disease? | Yes | 1 | H19 |
|  | No | 2 |  |

| **CORE: Lifestyle Advice** | | | |
| --- | --- | --- | --- |
| During the past three years, has a doctor or other health worker advised you to do any of the following?  *(RECORD FOR EACH)* | | | |
| Quit using tobacco or don’t start | Yes | 1 | H20a |
|  | No | 2 |  |
| Reduce salt in your diet | Yes | 1 | H20b |
|  | No | 2 |  |
| Eat at least five servings of fruit and/or vegetables each day | Yes | 1 | H20c |
|  | No | 2 |  |
| Reduce fat in your diet | Yes | 1 | H20d |
|  | No | 2 |  |
| Start or do more physical activity | Yes | 1 | H20e |
|  | No | 2 |  |
| Maintain a healthy body weight or lose weight | Yes | 1  *If C1=1 go to M1* | H20f |
|  | No | 2  *If C1=1 go to M1* |  |

| **Step 2 Physical Measurements** |
| --- |

| **CORE: Blood Pressure** | | | |
| --- | --- | --- | --- |
| **Question** | **Response** | | **Code** |
| Interviewer ID |  | └─┴─┴─┘ | M1 |
| Device ID for blood pressure |  | └─┴─┘ | M2 |
| Cuff size used | Small | 1 | M3 |
|  | Medium | 2 |  |
|  | Large | 3 |  |
| Reading 1 | Systolic ( mmHg) | └─┴─┴─┘ | M4a |
|  | Diastolic (mmHg) | └─┴─┴─┘ | M4b |
| Reading 2 | Systolic ( mmHg) | └─┴─┴─┘ | M5a |
|  | Diastolic (mmHg) | └─┴─┴─┘ | M5b |
| Reading 3 | Systolic ( mmHg) | └─┴─┴─┘ | M6a |
|  | Diastolic (mmHg) | └─┴─┴─┘ | M6b |
| During the past two weeks, have you been treated for raised blood pressure with drugs (medication) prescribed by a doctor or other health worker? | Yes | 1 | M7 |
|  | No | 2 |  |
| **CORE: Height and Weight** | | | |
| **For women:** Are you pregnant? | Yes | 1 *If Yes, go to M 16* | M8 |
|  | No | 2 |  |
| Interviewer ID |  | └─┴─┴─┘ | M9 |
| Device IDs for height and weight | Height | └─┴─┘ | M10a |
|  | Weight | └─┴─┘ | M10b |
| Height | in Centimetres (cm) | └─┴─┴─┘. └─┘ | M11 |
| Weight  *If too large for scale 666.6* | in Kilograms (kg) | └─┴─┴─┘.└─┘ | M12 |
| **CORE: Waist** | | | |
| Device ID for waist |  | └─┴─┘ | M13 |
| Waist circumference | in Centimetres (cm) | └─┴─┴─┘.└─┘ | M14 |

| **EXPANDED: Hip Circumference and Heart Rate** | | | | | | |
| --- | --- | --- | --- | --- | --- | --- |
| Hip circumference | | in Centimeters (cm) | | └─┴─┴─┘.└─┘ | | M15 |
| Heart Rate | | | | |  | |
| Reading 1 | Beats per minute | | └─┴─┴─┘ | | M16a | |
| Reading 2 | Beats per minute | | └─┴─┴─┘ | | M16b | |
| Reading 3 | Beats per minute | | └─┴─┴─┘ | | M16c | |

| **Step 3 Biochemical Measurements** |
| --- |

| **CORE: Blood Glucose** | | | |
| --- | --- | --- | --- |
| **Question** | **Response** | | **Code** |
| During the past 12 hours have you had anything to eat or drink, other than water? | Yes | 1 | B1 |
|  | No | 2 |  |
| Technician ID |  | └─┴─┴─┘ | B2 |
| Device ID |  | └─┴─┘ | B3 |
| Time of day blood specimen taken (24 hour clock) | Hours : minutes | └─┴─┘: └─┴─┘  hrs mins | B4 |
| Fasting blood glucose  *[CHOOSE ACCORDINGLY: MMOL/L OR MG/DL]* | mmol/l | └─┴─┘. └─┴─┘ | B5 |
|  | mg/dl | └─┴─┴─┘.└─┘ |  |
| Today, have you taken insulin or other drugs (medication) that have been prescribed by a doctor or other health worker for raised blood glucose? | Yes | 1 | B6 |
|  | No | 2 |  |
| **CORE: Blood Lipids** | | | |
| Device ID |  | └─┴─┘ | B7 |
| Total cholesterol  *[CHOOSE ACCORDINGLY: MMOL/L OR MG/DL]* | mmol/l | └─┴─┘. └─┴─┘ | B8 |
|  | mg/dl | └─┴─┴─┘.└─┘ |  |
| During the past two weeks, have you been treated for raised cholesterol with drugs (medication) prescribed by a doctor or other health worker? | Yes | 1 | B9 |
|  | No | 2 |  |
| **CORE: Urinary sodium and creatinine** | | | |
| Had you been fasting prior to the urine collection? | Yes | 1 | B10 |
|  | No | 2 |  |
| Technician ID |  | └─┴─┴─┘ | B11 |
| Device ID |  | └─┴─┘ | B12 |
| Time of day urine sample taken (24 hour clock) | Hours : minutes | └─┴─┘: └─┴─┘  hrs mins | B13 |
| Urinary sodium | mmol/l | └─┴─┴─┘.└─┘ | B14 |
| Urinary creatinine | mmol/l | └─┴─┘. └─┴─┘ | B15 |
